# Tamil Merchant in Ancient Mesopotamia

**DOI:** 10.1371/journal.pone.0109331

**Published:** 2014-10-09

**Authors:** Malliya gounder Palanichamy, Bikash Mitra, Monojit Debnath, Suraksha Agrawal, Tapas Kumar Chaudhuri, Ya-Ping Zhang

**Affiliations:** 1 Laboratory for Conservation and Utilization of Bioresources, Yunnan University, Kunming, China; 2 State Key Laboratory of Genetic Resources and Evolution, Kunming Institute of Zoology, Chinese Academy of Sciences, Kunming, China; 3 Cellular Immunology Laboratory, University of North Bengal, Raja Rammohanpur, Darjeeling, India; 4 Department of Medical Genetics, Sanjay Gandhi Institute of Medical Sciences, Lucknow, India; University of Florence, Italy

## Abstract

Recent analyses of ancient Mesopotamian mitochondrial genomes have suggested a genetic link between the Indian subcontinent and Mesopotamian civilization. There is no consensus on the origin of the ancient Mesopotamians. They may be descendants of migrants, who founded regional Mesopotamian groups like that of Terqa or they may be merchants who were involved in trans Mesopotamia trade. To identify the Indian source population showing linkage to the ancient Mesopotamians, we screened a total of 15,751 mitochondrial DNAs (11,432 from the literature and 4,319 from this study) representing all major populations of India. Our results although suggest that south India (Tamil Nadu) and northeast India served as the source of the ancient Mesopotamian mtDNA gene pool, mtDNA of these ancient Mesopotamians probably contributed by Tamil merchants who were involved in the Indo-Roman trade.

## Introduction

The origin and civilization of people living in ancient Mesopotamia has been the subject of intense debate over the past several decades. Recently skeletal remains dated between Early Bronze Age and the Late Roman period (between 2500 BCE and 500 AD) were excavated from two archaeological sites located in the middle Euphrates valley. Witas and colleagues [1] have analyzed the mitochondrial DNA (mtDNA) obtained from those skeletal remains and argued that the people living in ancient Mesopotamia carried mtDNA haplotypes corresponding to the M65a, M49 and/or M61haplogroups. Importantly, these mtDNA haplogroups are commonly found in the populations belonging to the Indian subcontinent, however, are absent in people living in Syria. This study has suggested that the skeletal remains from Mesopotamia belonged to people who have genetic affinity with the populations living in the Indian subcontinent. This observation has raised a pertinent question as whether M65a, M49 and/or M61 haplogroups carrying individuals were descendants of ancient migrants who might have founded regional Mesopotamian groups like that of Terqa or they were the merchants who used these region for trading. To address these issues, the present study was performed more comprehensively on a large mtDNA data set. The findings of our study clearly support the later proposition, suggesting that M65a, M49 and/or M61 haplogroups carrying ancient Mesopotamians might have been the merchants from India.

## Materials and Methods

To identify the ancient Mesopotamian mitogenome lineage in the Indian subcontinent, we tested about 4,319 unrelated individuals from the Indian subcontinent. All DNA samples analyzed in the present study were derived from the blood samples collected between 2001 and 2004. The authors obtained written informed consent from the volunteers or community leaders after explaining the collection procedures and purpose of the study in local languages. The institutional Ethical Committees of North Bengal University, Sanjay Gandhi Institute of Medical Sciences and the Yunnan University approved the protocol and ethical clearance of the study.

The mtDNA sequence data obtained from 4,319 individuals represent 1,348 south Indians (from Andhra Pradesh, Kerala and Tamil Nadu), 568 north Indians (from Uttar Pradesh), and 2,403 east/northeast Indians (from Jharkhand, Meghalaya, Sikkim, and West Bengal) as well as people from adjoining areas of Nepal, Bhutan, and Bangladesh. The hypervariable segment of the mtDNA control region (HVS-I and HVS-II) and the haplogroup defining coding region were amplified and sequenced in all studied individuals. The mtDNA haplogroups were classified using the HVS-I and HVS-II variable sites and the diagnostic variants in the coding regions were identified based on the up-to-date mtDNA phylogenetic tree (Phylotree.org, build 16, February 19, 2014) [2].

### Control region data mining

The mtDNA control region-I matches for the ancient Mesopotamian MK 13G 117, TQ 28F 112, TQ 28F 256 and MK 11G 107 individuals were searched from the South Asian published datasets; see Tables S1 and S2 in File S1 for further details and references. We screened a total of 15,751 mtDNA genomes (11,432 from the literature and 4,319 from this study) representing all major populations residing across India (Table S2 in File S1).

### SNP typing

To identify the precise phylogenetic relationship of the ancient Mesopotamian bearing Indian-specific M49c and M65a lineages, we screened several SNPs (511, 1664, 2177, 4655, 4721, 4916, 5302, 6261, 6584, 8251, 9254, 11413, 12635, 13923, 13651, 15924 and 15930) in two haplotypes, which share control region motifs with the ancient Mesopotamian lineage- MK 11G 107. These SNPs were chosen from the published whole mtDNA haplogroup M65a genome data [2], [3]. In addition, four M49c and two M65a samples were chosen for complete sequence analysis. DNA sequencing was carried out as described in Palanichamy et al. [4]. Mutations were scored relative to the revised Cambridge Reference Sequence (rCRS) [5].

### Phylogenetic tree, network, and molecular dating

Maximum parsimony trees were built for complete M49c and M65a genomes and HVS-I segments. The coalescent times of M49c and M65a lineages were estimated with rho- statistics (ρ) (mean divergence from inferred ancestral haplotype) [6], and standard errors (σ) were calculated following the method of Saillard et al. [7]. The calculator provided by Soares et al. [8] was used to convert the ρ-statistics and its error ranges to age estimates with 95% confidence intervals.

## Results and Discussion

It seems difficult to identify the exact source population from Indian subcontinent that contributed to the mtDNA signatures of the ancient Mesopotamian MK 13G 117, TQ 28F 112, and TQ 28F 256 individuals based only on the mtDNA hypervariable control region matches, as fast mutation rate within this region can lead to similarities due to homoplasy [9]. The haplogroup assignments used for the ancient Mesopotamian samples- MK 13G 117, TQ 28F 112, and TQ 28F 256 by Witas et al. [1] are based on HVS-I sequences alone, this can lead to serious inaccuracies in data interpretation. For instance, HVS-I sequence exhibiting transition at 16223 and 16234 in sample TQ 28F 112 sample may belong to either M30 or M49 (Table 1). Further, the HVS-I sequence of MK 13G 117 differs from the Cambridge reference sequence [5] at 16223, 16234 and 16311 and may belong to either M49 or M9a (Table 1).

**Table 1:** mtDNA haplotype affiliated with the ancient Mesopotamian and related sequences in the Indian subcontinent.

Region	Id	HVS-I (16000+)	HVS-II (73 and 263 in addition)^a^	Haplogroup	References
					
Ancient Mesopotamia	TQ 28F 112	223-234	NA	M49	[1]
India West Bengal	SW2, Ra64, RJ133	223–234	195A	M30	Present study
Uttar Pradesh	C47, C199, C186	223–234	195A	M30	Present study
Delhi	Hindu	223–234	195A	M30	[9]
Tamil Nadu	TM42, TM44	223–234	195A	M30	[10]
West Bengal	Mun223, DH3, DH22, ME30, BO23/2, MX52/4	223–234	204–249del	M49c1	Present study
Bangladesh	Chakma292, Tripura13	223–234		M49c1	[11]
Myanmar	MMR019	223–234		M49e1	[12]
Assam	HC10	223–234		M49c1	[13]
China Tibet	DXT971, LZG079	223–234		M49c1	[14]
Ancient Mesopotamia	TQ 28F 256	223–234–270	NA	M49 or M61	[1]
					
India Assam	Ahom 17	38–192–223–234–270		M49c1a	[13]
West Bengal	Ra23, Sc33, Ra44, RJ221, Ra69	38–192–223–234	152–195	M49c1a	Present study
Assam	SK16, SK112	38–192–223–234	152–195, 152–195–198	M49c1a	[15]
Ancient Mesopotamia	MK 13G 117	223–234–311	NA	M49	[1]
India Odisha	PB23	223–234–311		M49c1	[15]
Bangladesh	Chakma267, 268	223–234–311		M9a	[11]
Myanmar	MMR049	223–234–300–311	146–195	M49e1	[12]
Ancient Mesopotamia	MK 11G 107	223–266–289		M65a	[1]
India Tamil Nadu-Dindigul	MX5, VEM17	223–266–289		M65a	Present study
	VKD32, DG43	223–266–289–311		M65a	
	NAT10	223–266–289–319		M65a	
	ODC28, KAM22, ARA18	209–223–266–289		M65a	

Mutations are scored relative to the rCRS [4]. ^a^Length variation in the 303–309 and 311–315 C-stretch region is ignored.

**Figure 1:**
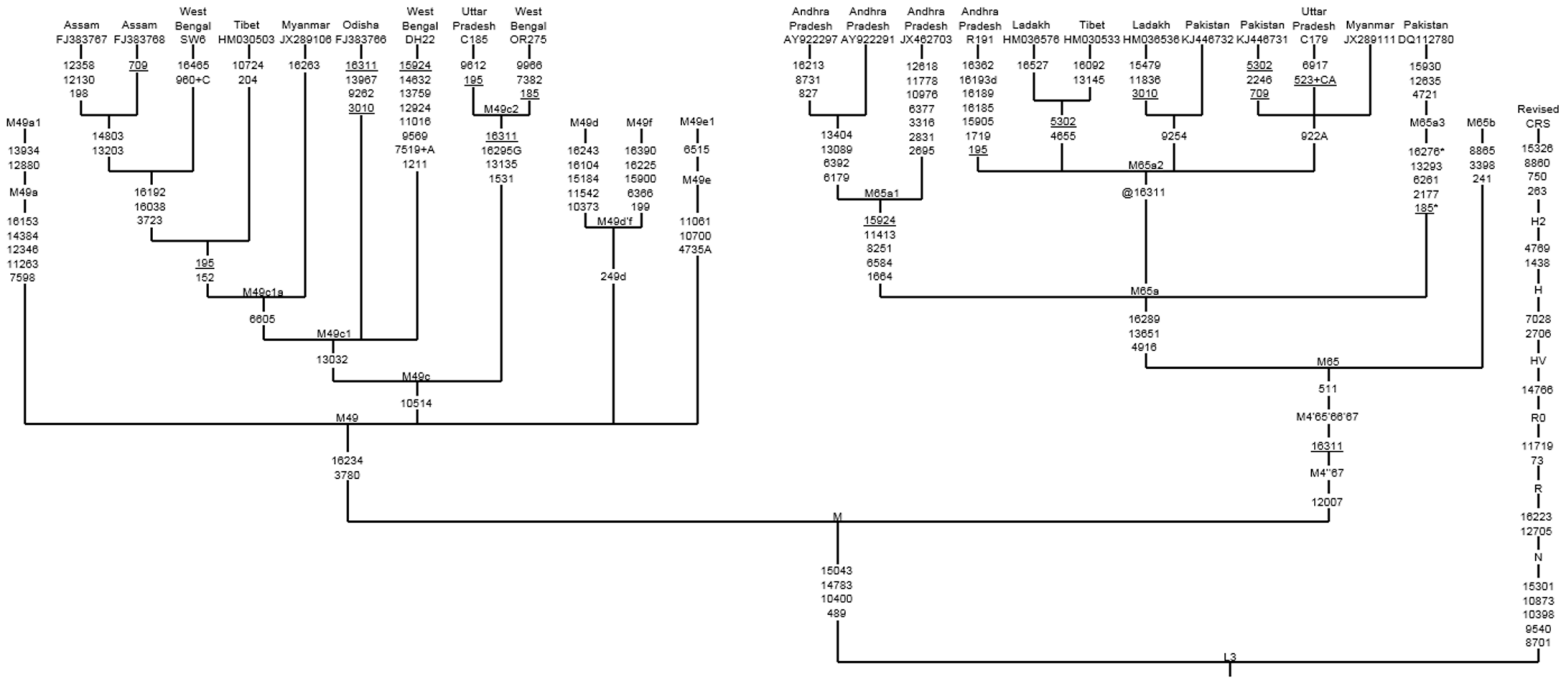
Maximum parsimony tree of entire mtDNA genomes belonging to ancient Mesopotamian haplogroups M49 and M65. Mutations are scored relative to the revised Cambridge reference sequence (rCRS) [5] and displayed along the branches. The prefix “@” indicates back mutation, recurrent mutations are underlined, transversions have a base suffix, “d” deletions and “+” insertions, and the poly(C) region in HVS1 and -2 as well as 16519 is excluded. The geographic origin of the sample and the accession number which is retrieved from the publication are denoted on top of the branches.

It is noteworthy that MK 13G 117, TQ 28F 112, and TQ 28F 256 sequences have some linked polymorphic sites such as 16223 and 16234, indicating that they are likely the descendants of South Asian mtDNA haplogroup M49 (Table 1). The complete genome tree shows that M49 has five main subclades (M49a, M49c, M49d-M49f), with M49d and M49f tentatively united by a deletion in control region position 249 to form the putative subclade M49d'f. Based on the M49 subclade control-region information, we noticed that the ancient Mesopotamian MK 13G 117, TQ 28F 112 and TQ 28F 256 lineages were belonged to subhaplogroup M49c (Fig. 1). We further sequenced four mtDNA genomes belonging to this haplogroup and identified a new variant, and revised the nomenclature as M49c as M49c1. Now the revised phylogenetic tree labels 10514 as the basal mutation for M49c subhaplogroup [2]. In addition, our sequence data led us to identify another additional subclade designated as M49c2 that has not been reported earlier. The refined phylogenetic analysis based on the above observations suggests that the ancient Mesopotamian MK 13G 117, TQ 28F 112 and TQ 28F 256 lineages might belong to subhaplogroup M49c1 and M49c1a (Fig. 1). The estimated date for the most recent common ancestor of M49c lineages descended from M is 21.9 kya (95% C.I.: 5.0-38.7).

**Figure 2:**
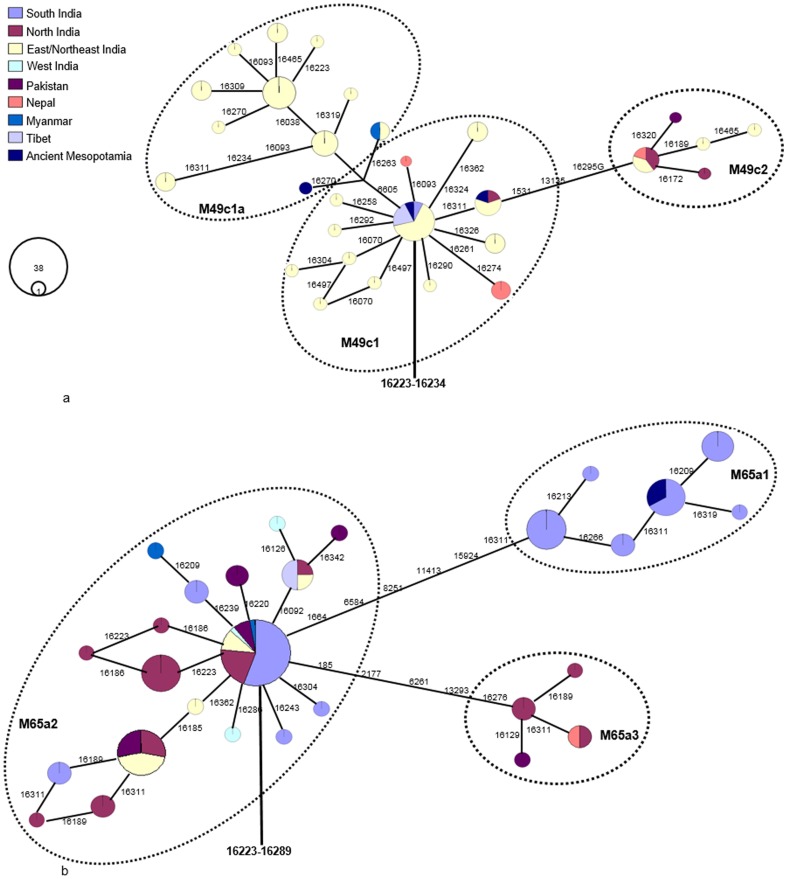
Maximum parsimony tree of available mtDNA control region sequences related to ancient Mesopotamian haplotypes. (**A**). Depict the east/northeast Indian origin of the ancient Mesopotamian haplotypes- MK 13G 117, TQ 28F 112, and TQ 28F 256. (**B**). Representing the ancient Mesopotamian- MK 11G 107 related haplotype distribution in the south India region. Circle areas are proportional to haplotype frequencies. All sequences were retrieved from the published data (see Table S1 for more information).

The HVS-I motif 16223-16234, 16223-234-270, 16223-234-16311 of haplotype M49c1 and M49c1a present in the ancient Mesopotamian samples- MK 13G 117, TQ 28F 112, and TQ 28F 256 are predominantly found in the eastern/northeastern Indians (from Odisha, West Bengal, Assam, Arunachal Pradesh, and Tripura) as well as populations from adjoining Bangladesh, Myanmar, and Nepal (Fig. 2a; Table S1 in File S1). Interestingly, the trade relations between India and Mesopotamia during the third millennium BCE and India played a crucial role in supplying timber for shipbuilding, mostly teak wood from the northeast and Myanmar [10]. During the trading over a long period of time, it might be possible that some of the northeast India traders traveled to Mesopotamia and contributed M49c1 lineages in the ancient Mesopotamian gene pool.

**Figure 3:**
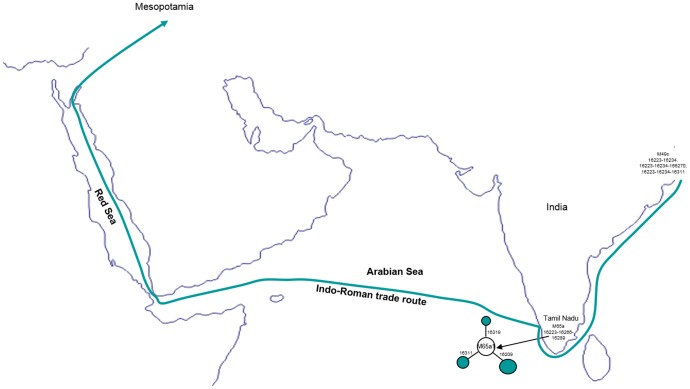
Ancient Indo-Roman maritime trade route. The geographic localization of ancient Mesopotamian mtDNA lineages M49c and M65a origin.

Unlike the MK 13G 117, TQ 28F 112, TQ 28F 256 samples, the mitogenome sequence of ancient MK 11G 107 individual exhibited substitutions in the control as well as diagnostic coding region sites (73-489-511-10398-10400-16223-16266-16289), this indicates that this individual might have originated from Indian sub-haplogroup M65a origin [2]. It is evident that the data obtained from the M65a mitogenomes available in the literature and databases (Table S1 and S2 in File S1) and our SNPs data from two haplotypes, share control region motifs with the ancient Mesopotamian lineage- MK 11G 107, this further implies that MK 11G 107 lineage belongs to subhaplogroup M65a1 (Fig. 1; Table S1 in File S1). Besides, five different mtDNA M65a haplotypes detected by Witas et al. [1] (see their Figure 1b) were classified now into two subhaplogroups- M65a2 and M65a3 (Fig. 2b; Table S1 in File S1). M65a2 lineage is widely distributed across India, Apart from India, it is also found in Pakistan, Myanmar and Tibet populations. Haplotype M65a3 has been observed in populations of northern India, Nepal and Pakistan (Table S1 in File S1). On the contrary, M65a1 is exclusively found in the south India (Fig. 2b). Fifteen M65a1 HVS-I haplotypes were observed, of which seven were in Andhra Pradesh/Telangana and eight were in Tamil Nadu. M65a1 could have originated in south India and the local divergence dates back to 31.5 kya (95% C.I.: 8.5–54.5). It is intriguing to note that the precisely matched sequence of ancient Mesopotamian –MK 11G 107 haplotype (16223-16266-16289) was observed in the populations living in a restricted area of Dindigul district of Tamil Nadu, India (Table 1 and Table S1 in File S1). Historical documentation indicates that during first centuries AD, the trade between Rome and south India got intensified; pearls, ivory, textiles and gold ornaments were imported from Tamil Nadu of south India- and Tamil merchants actively engaged in the overseas trade with Rome [11]. In addition, earlier archaeological evidence (pots with Tamil-Brahmi inscriptions found in Quseir-al-Qadim, an ancient port with a Roman settlement on the Red Sea coast of Egypt) supports the hypothesis of trade between Tamil and Rome [12]. The affinities of ancient MK 11G 107 mtDNA with south Indian Tamilians and its absence in other region suggests that this individual may have originated from south India and might have a Tamil connection (Fig. 3).

Taken together, our mtDNA analysis shows that mtDNAs of these ancient Mesopotamians probably originated from Indian merchants. This, thus rules out the hypothesis that these samples comprise an ancient component (Upper Paleolithic) of macrohaplogroup M involved in the founding of the Mesopotamian civilization. Therefore, the present study sheds new insights on the understanding of the origins of ancient Mesopotamian macrohaplogroup M lineages and the influence of Indian-Tamil merchants to Mesopotamian gene pool during trans-oceanic trade.

## Supporting Information

File S1 **This file contains Table S1 and Table S2.** Table S1, Distribution of M49 and M65a haplotypes. Table S2, South Asian samples used in the present study.
(XLS)
